# Needs and unmet needs for support services for recently pregnant intimate partner violence survivors in Ethiopia during the COVID-19 pandemic

**DOI:** 10.1186/s12889-023-15634-7

**Published:** 2023-04-20

**Authors:** Robel Yirgu, Abigiya Wondimagegnehu, Jiage Qian, Rachel Milkovich, Linnea A. Zimmerman, Michele R. Decker, Nancy Glass, Fatuma Seid, Lensa Zekarias, Shannon N. Wood

**Affiliations:** 1 Addis Ababa University School of Public Health, Addis Ababa, Ethiopia; 2 Department of Population Family and Reproductive Health, Johns Hopkins Bloomberg School of Public Health, Baltimore, MD USA; 3 Center for Public Health and Human Rights, Johns Hopkins Bloomberg School of Public Health, Baltimore, MD USA; 4 Johns Hopkins School of Nursing, Baltimore, MD USA; 5 Center for Global Health, Johns Hopkins University, Baltimore, MD USA; 6 Ethiopia Federal Ministry of Health, Addis Ababa, Ethiopia

**Keywords:** Intimate partner violence, Services, Resources, COVID-19, Violence response, Disclosure, Ethiopia

## Abstract

**Background:**

Globally, 2–14% of women experience intimate partner violence (IPV) during pregnancy. Timely response to IPV is critical to mitigate related adverse health outcomes. Barriers to accessing limited IPV support services are pervasive in low- and middle-income countries (LMICs), such as Ethiopia; key barriers include mistrust, stigmatization, and self-blame, and discourage women from disclosing their experiences. Infection control measures for COVID-19 have the potential to further disrupt access to IPV services.

**Methods:**

In-depth qualitative interviews were undertaken from October-November 2020 with 24 women who experienced IPV during recent pregnancy to understand the needs and unmet needs of IPV survivors in Ethiopia amid the COVID-19 pandemic. Trained qualitative interviewers used a structured note-taking tool to allow probing of experiences, while permitting rapid analysis for timely results. Inductive thematic analysis identified emergent themes, which were organized into matrices for synthesis.

**Results:**

Qualitative themes center around knowledge of IPV services; experiences of women in seeking services; challenges in accessing services; the impact of COVID-19 on resource access; and persistent unmet needs of IPV survivors. Notably, few women discussed the violence they experienced as unique to pregnancy, with most referring to IPV over an extended period, both prior to and during COVID-19 restrictions. The majority of IPV survivors in our study heavily relied on their informal network of family and friends for protection and assistance in resolving the violence. Though formal IPV services remained open throughout the pandemic, restrictions resulted in the perception that services were not available, and this perception discouraged survivors from seeking help. Survivors further identified lack of integrated and tailored services as enduring unmet needs.

**Conclusions:**

Results reveal a persistent low awareness and utilization of formal IPV support and urge future policy efforts to address unmet needs through expansion of services by reducing socio-cultural barriers. COVID-19 impacted access to both formal and informal support systems, highlighting needs for adaptable, remote service delivery and upstream violence prevention. Public health interventions must strengthen linkages between formal and informal resources to fill the unmet needs of IPV survivors in receiving medical, psychosocial, and legal support in their home communities.

## Background

Intimate partner violence (IPV) - physical, sexual, or emotional abuse perpetrated by an intimate partner – adversely impacts women’s reproductive, sexual, and mental health [1–3]. IPV is pervasive globally; approximately one in three women experience IPV over the course of their lifetime [2, 4]. IPV during pregnancy, though less prevalent (2–14%) [5, 6], is of particular concern given its acute health impact to mother and baby over a short period of time, with consequences including miscarriage, premature labor, low birthweight, and maternal depression [7–10]. Research indicates that while pregnancy is generally not a point of initiation for IPV, pre-existing relationship tension can heighten during pregnancy, leading to increased IPV frequency and severity [6]. Further, abusive partners may limit access to health services, including antenatal and postnatal care [6]. Given the severity and potential health effects, timely access to services is critical [5, 10].

Pregnancy and the postpartum period are potentially pivotal timepoints for health system response to violence, given multiple points of contact with providers [11, 12]. Connecting IPV survivors to services is critical for health and safety, and counseling on safety strategies may decrease subsequent violence and increase safety [10]. Globally, however, key barriers remain in accessing IPV support services—these barriers are pervasive in low- and middle-income countries (LMICs) where communities normalize violence as a familial issue and stigmatize women for help-seeking, and where limited violence response services exist [13–15]. Mistrust, stigmatization, and self-blame may lead women to seek help only from their most trusted sources of support, with little engagement with more formal health structures [16, 17].

Ethiopia is one such context where women suffer a severe IPV burden but rarely seek help for the violence they experience. Recent Ethiopia Demographic and Health Survey data indicate that 27% of ever-married women age 15–49 have experienced IPV within the past year, and 4% ever experienced IPV during pregnancy [18]. Other sources, primarily hospital-based, estimate that the prevalence of IPV during pregnancy may be as high as 45% [19–21]. Moreover, large-scale surveys likely underestimate the true burden of IPV [22]. High prevalence is amplified by significant gaps to understanding women’s disclosure of IPV experiences and utilization of IPV services. More than two-thirds of IPV survivors in Ethiopia never disclosed or sought help, and less than 10% sought help from formal services [18, 23].

With the spread of the COVID-19 pandemic, there is concern that infection control measures, such as social distancing and stay at home orders, may contribute to heightened levels of violence [24, 25]. Additionally, indirect effects of the pandemic, such as loss of income, strained social support, and closures of violence response services, may further exacerbate IPV and help-seeking [25–27]. Several studies have examined the impact of the COVID-19 pandemic on women’s health services in LMICs [28–30], however, few have sought to understand IPV needs and unmet needs from the woman’s perspective. The purpose of the current study is to understand the needs and unmet needs of IPV survivors in Ethiopia during the COVID-19 pandemic through in-depth qualitative interviews. Our findings elucidate ways to fill these gaps to reduce unmet needs and sustain IPV support during crisis situations. While the present study objectives were specific to the COVID-19 pandemic, notably, many of the unmet needs discussed by IPV survivors pre-date the COVID-19 pandemic and therefore, a much broader discussion of women’s unmet needs for IPV is presented.

## Methods

### Study design

This qualitative study is situated within the Performance Monitoring for Action (PMA)-Ethiopia cohort study, a collaboration between Johns Hopkins Bloomberg School of Public Health (JHSPH), Addis Ababa University (AAU), and the Ethiopian Federal Ministry of Health (FMoH). PMA-Ethiopia collects data on a cohort of 2,879 pregnant women at pregnancy, 6-weeks, 6-months, and 1-year postpartum. Enrollment into the cohort began in October 2019. The full protocol for PMA Ethiopia is detailed elsewhere [31].

A State of Emergency in Ethiopia was declared in response to COVID-19 approximately halfway through fielding the 6-week postpartum interview (April 8, 2020). Specifically, 1,405 6-week interviews were conducted pre-COVID-19; the remaining 983 occurred after COVID-19 emergency lockdown procedures eased in early June. Following the 6-week postpartum interviews that occurred after the COVID-19 emergency lockdown procedures ceased, in-depth interviews contextualized experiences of IPV during pregnancy, among a subset of participants from Oromiya and Southern Nations, Nationalities, and Peoples’ Regions (SNNP; n = 24 interviews total). These regions were selected based on highest baseline prevalence of IPV during pregnancy and feasibility.

### Study procedures

The qualitative study followed all quantitative data collection. At the 6-week postpartum surveys conducted after COVID-19 emergency lockdown procedures ceased, women who indicated any experience of IPV during pregnancy per behavioral assessment (Revised Conflict and Tactics Scale) [32], were asked if they were interested and consented for follow-up interview.

A purposive sampling frame was used to select participants from 6-week postpartum survey data, with inclusion criteria specifying: (1) completion of quantitative 6-week post-COVID interview; (2) indication of IPV experience via quantitative data; (3) consent to be followed up for qualitative interview.

Interviews were conducted from October to November 2020, with participants called prior to interview for scheduling considerations. Interview guides focused on partner relationships and IPV experiences both prior to and during the COVID-19 pandemic, as well as prior to, during, and after pregnancy. The remainder of the interview guide focused on IPV response, informal and formal discussions, and availability of and helpfulness of services; all questions probed on changes in service provision, access, and quality with the COVID-19 pandemic. A separate four-person trained qualitative data collection team conducted the qualitative interviews, however, the original quantitative data collector joined the qualitative interviewer to help identify the woman’s location and identity, as well as explain study procedures. At this time, all participants were again asked if they would like to participate, and full oral consent procedures were conducted. All interviews lasted approximately 25–30 min. Semi-structured interview guides focused on women’s experiences with IPV and IPV services. Trained qualitative interviewers (two per interview) used a structured note-taking tool to allow probing of experiences, while permitting rapid analysis for timely results [33]. Data collection continued until feasible sample size was met (n = 14 Oromiya; n = 10 SNNP) [34]. Immediately post-interview, interviewers typed and translated field notes.

### Ethical procedures

Training for the qualitative phase preceded data collection with focus on probing, ethical principles for IPV research, and research team protections. All women were offered warm referrals, or interviewer facilitated referrals, to local support services, including health centres equipped with violence and psychosocial support. Institutional Review Board approval for human subjects research, inclusive of oral consent procedures, was obtained at both JHSPH (IRB00013278) and AAU College of Health Sciences (077/20/SPH), and protocols were implemented in line with best practices for violence research [35].

### Analysis

Two researchers trained in qualitative analysis coded 24 structured notes using Atlas.ti software. Inductive thematic analysis was used to identify emergent themes and sub-themes and to create an initial set of codes; the coders completed this process separately and then joined together to compile the initial codebook. Dual coding (concurrent, separate coding by the two qualitative researchers) and retro-coding (redoing the coding should disagreements occur) were used to enhance agreement between coders and credibility of results, with coders meeting after every two transcripts to assess agreement and revise as needed. Coders also met and collaborated weekly with the field research team to discuss and clarify interpretation. Coding was complete when saturation of themes was achieved [36]; illustrative quotes were then downloaded from Atlas.ti and organized in matrices of code sub-themes, which were later aggregated into larger themes based on commonality of sub-themes.

## Results

Background characteristics of respondents are presented in Table 1. Five emergent themes were identified from the data: (1) knowledge of IPV services; (2) experiences of women in seeking IPV services; (3) challenges to accessing IPV services; (4) the impact of COVID-19 pandemic on access to and utilization of IPV services; and (5) persistent service needs for women experiencing IPV. While the intention was initially to explore the influence of COVID-19, results indicated that perceived barriers and unmet needs largely pre-dated COVID-19.

**Table 1:** Background characteristics of respondents

	Total (N = 24)
**Age, mean (sd)**	26.8 (7.5)
**Region, n (%)**	
Oromiya	14 (58%)
SNNP	10 (42%)
**Residence, n (%)**	
Rural	19 (79%)
Urban	5 (21%)
**Education, n (%)**	
None	7 (29%)
Primary	13 (54%)
Secondary or higher	4 (17%)
**Religion, n (%)**	
Protestant	9 (38%)
Orthodox	2 (8%)
Muslim	13 (54%)
**Marital status, n (%)**	
Married	22 (92%)
Living with a partner	2 (8%)
**Parity, n (%)**	
0	6 (25%)
1	6 (25%)
2	4 (17%)
3	8 (33%)

### Knowledge of IPV services

Women had high awareness of informal IPV supports, and many shared experiences of seeking help from their neighbors, family members, and friends. Community elders were often viewed as reliable sources in helping couples navigate relationship disagreements.

… if a woman experiences partner violence, she will go to her neighbors. Or if it’s possible, the community elders will manage the conflict. – IPV survivor, Age 23, Oromiya

For some women, these were the only sources of support known within the community.

I only know about traditional services given by village elders and church leaders. Village leaders negotiate with husband and wife. – IPV survivor, Age 25, SNNP

For others, they acknowledged that formal IPV supports were available locally, but most did not know details on how to access or the specific types of services.

…there is women’s affairs office in our area, but I don’t know what kind of services they give. I only know women who faced gender violence seek their services. Otherwise, I don’t have detailed information on the type of service they provide. – IPV survivor, Age 18, Oromiya

### Experience of women in seeking IPV services

Overall, informal sources from family members, neighbors, and village elders appeared to be the first resources women turned to for IPV support. The majority of women discussed that these support systems were useful for encouraging negotiations between partners, as well as offering temporary housing as a way to de-escalate the situation.

When such kind of things happen, I immediately go to my neighbors. He has a brother in town, and I inform him what he said to me and warn him as I will go back to my parents’ house. Then, he advises him and try to negotiate us. – IPV survivor, Age 25, Oromiya

When I had a disagreement with my husband and left home, my neighbors followed me to my parents and asked me why I left my house. At that time, I told them everything. Then the elders back at our home, they gave advice to my husband. After the elders advised him, he stopped blaming the children. There is a change regarding to our relationship. – IPV survivor, Age 37, Oromiya

I went to them (community elders) once during my first pregnancy when my partner had hit me and even threatened me. I went to them and told them what he had done to me and they told me that they would speak to him and make him stop what he is doing to me. After they spoke to him, he agreed to not do it… After that incident, he hasn’t beaten me and the physical abuse has stopped. – IPV survivor, Age 22, Oromiya

These informal sources of support were not always helpful, and many women disclosed that community elders and village leaders advised them to tolerate rather than act on the abuse.

Even if I wanted to go to the police station, the village elders didn’t allow us to go there or would keep us from going to the police station. It’s not acceptable to go to the police station in this community. The village elders wouldn’t accept this kind of behavior. It is believed in our community that if someone gets married, they cannot get divorced or go to the police station. Your only choice is to tolerate each other. – IPV survivor, Age 42, SNNP

One participant further spoke of poor judgement within the village elder system.

There are village leaders here but they’re useless. They take bribes and they want the woman to just sit and take it. So, I never talk to them. – IPV survivor, Age 30, SNNP

Formal services, on the other hand, were a last resort for women experiencing IPV. As indicated by many, women only sought help from the police (for partner arrest, filing for divorce, and negotiation), court (for suing the husband/partner, issuing sentences, processing a divorce, and negotiation), health centers (for treatment and sometimes medical certificate), or other formal IPV support avenues, when informal support systems could not resolve the issue. It should be noted that many sought out formal services with the intention of stopping the violence and in hope of keeping their marriage intact, rather than ending it, to avoid social and financial repercussions. Legal services (police/court) were sought as the last option while women navigated gradually through support systems and remained cautious about protecting their marriage while doing so.

Getting a divorce isn’t an appropriate thing to do in our community. But if there is a problem that’s above the capacity of village leaders like death threats and the like...am not saying there are any situations like that though. In such case, they can get help from police and health treatment from health center. – IPV survivor, Age 30, SNNP

Women don’t want to take their case to a legal body. Here, if a woman experienced violence, the first step will be telling her neighbors. Then, if it is impossible to solve the problem, she will inform her friends. But if it is hard and complicated at this level, she can take the case to the court. Women prefer to tolerate the violence in their whole life. – IPV survivor, Age 18, Oromiya

In cases of severe violence, women indicated they were able to obtain some relief after seeking help from legal services.

I was very hopeless and wanted to die when he hits me while I was eight months pregnant. Then I begged the police to divorce us and told him as I want to live for my children. I also shared my concern as he might kill me if I return to my home…The police tried to calm me down and I returned home. But he (husband) stayed for two days in prison and signed a letter as he would never hit me again. – IPV survivor, Age 34, Oromiya

After the police arrested him (husband), he became a good person and I start to live as human being. Before that, I was living in darkness; I was crying day and night. My life was full of sorrow. Now, I came out of darkness and feel like I am born again. – IPV survivor, Age 34, Oromiya

### Challenges to accessing IPV services

Long distance to services and limited availability of formal services at the kebele level hindered women’s access to adequate IPV support. Complimentary to the immediate actions offered by the police or court, women’s affairs offices provide social and psychological support to women who decided to seek legal support and help them prevent future violence. Unfortunately, services from women’s affairs offices and health facilities seemed particularly limited at the local level; one woman stated that it took up to four hours to access the closest women’s affairs office. These barriers disproportionally impacted women who could not afford transportation.

There are community police, but the police station is a bit far. Besides, there is no women’s affairs office—we don’t have that service here. Therefore, if one woman experiences partner violence, and if the case is complicated, she needs to go to women’s affairs office which is only available at the woreda level (also known as district, or third-level administrative divisions). – IPV survivor, Age 23, Oromiya

There is no police station here in our locality and I didn’t even hear about women’s affairs office. There is a health facility, but I don’t know whether they are working on this issue.” – IPV survivor, Age 17, Oromiya

Actually, we have kebele (or neighborhood level) to report this case. But most of the [health] services are found at the woreda level. Whenever there is partner violence, women might not have money to cover their transportation cost. I mean, even if they want to go to town, they may not have 10 ETB (0.23 USD) for transportation. So, they continue living their life with all those challenges.” – IPV survivor, Age 25, Oromiya

Tolerance of violence due to economic dependence was common. Women with no or insufficient income were afraid to report IPV to legal bodies, as they would not have enough money to raise their children if their relationship ended in divorce or separation.

The main challenge is associated with income. If women don’t have sufficient income, they are afraid to take their case to the court. Because they don’t want to get divorced as they don’t have enough money to raise their children. – IPV survivor, Age 37, Oromiya.

Fear of familial separation and need to preserve the family unit further served as a deterrent for women to seek formal services.

Women prefer to raise their children no matter what violence they have. They will tolerate the violence in their whole life… – IPV survivor, Age 18, Oromiya.

Especially, if she has children and no income; how can she raise her children? So, she tolerates every pain for the sake of her children. – IPV survivor, Age 29, Oromiya.

Furthermore, some women were fearful that their partners would get angry and abuse them even more if they reported their cases to formal resources, like the police. This suggested a need for formal IPV services to not only focus on an immediate resolution but also ensure women’s safety while seeking services.

If a woman reports the case to the police, her husband may harm her; if she has no family around, she can’t go anywhere after accusing him. So, she tolerates the situation. – IPV survivor, Age: 29, Oromiya.

The other major challenge for not seeking the service is fear of their husband. Women might think, if I take our case to a legal body, he doesn’t necessarily stop the violence. – IPV survivor, Age 18, Oromiya.

The social environment discouraged women from seeking IPV protection in several ways. First, women experienced victim-blaming when discussing IPV, which was most often mentioned while accessing informal services. This lack of validation when sharing something extremely vulnerable served as a major barrier for future help-seeking.

I told my brother how my husband beat me; he didn’t trust me, he even said it’s my fault that he beat me.... Because no one was going to take my side and protect me, I decided not to tell anyone and seek help from others. – IPV survivor, Age 42, SNNP

People talk a lot around here and gossip behind your back. Because of that, I don’t want to tell anyone. There is a woman who works here with me so sometimes we talk. I tell her that he is angry today and she advises me, “Leave it to God and just focus on your kids”. – IPV survivor, Age 30, SNNP

Lastly, women described the high societal value placed on maintaining a marriage. Exposing stories of IPV and revealing betrayal between partners are taboo topics even within families, let alone the community at large. These norms led many women to remain silent about the abuse they were enduring.

I haven’t involved anyone so far. I can’t talk about my personal life with anyone…even my family. This is my problem so no one can help me. – Age: 38, SNNP

### Impact of COVID-19 pandemic on access to IPV resources

Pre-existing access difficulties were exacerbated by the COVID-19 pandemic. Women discussed both direct service disruptions and women’s perceptions about service closures due to the pandemic and related mitigation measures.

When coronavirus pandemic occurred, every sector was closed like police and women’s affairs office except health centers. – IPV survivor, Age 18, Oromiya

COVID has an impact on the availability of the service. People were talking a lot about its effect, and I heard that the police station was closed. I also wanted to go to the police twice but changed my mind assuming there might not be service during this time. – IPV survivor, Age 25, Oromiya

Of note, it was unclear whether women attempted to access these services during COVID-19 restrictions, or whether there was a perception that these services were closed.

Additionally, many mentioned the increase in transportation costs as a factor that placed barriers on women attempting to seek formal IPV services during COVID-19.

All organizations like women’s affairs office, police and court are in the town so, it is difficult to go there since the transportation cost increased during this coronavirus pandemic. – IPV survivor, Age 28, Oromiya

While formal service utilization was impacted by the few attempting to seek these services, difficulties attempting to access informal services were far greater. Specifically, public health control measures such as social distancing, stay home orders, and restrictions of on movement hindered women from receiving informal IPV supports from neighbors, family, friends, and religious leaders that they usually sought pre-pandemic.

Before coronavirus I used to tell the mosque Imam and village elders when we have disagreements. But after coronavirus, there were movement restriction, and I could not meet anyone. – IPV survivor, Age 36, SNNP

…But after COVID, things are totally changed. I am worried how to discuss with family and friends if something happens to me. Because no one goes to others house and most of the time we were staying in our home. Even if you face any violence during this time, it is forbidden to get advice from friends and neighbors. – IPV survivor, Age 23, Oromiya

### Unmet needs of IPV survivors

Foremost, IPV survivors described a need to better understand the types of services offered at each facility in order to help them make the best choices for their situation and navigate these services. Clear guidelines on services offered could further assist in weighing benefits of services versus access and cost related drawbacks.

Some women don’t have awareness on this. The other women didn’t believe in its benefit. Women might think, I don’t get any solution from getting this service… People’s understanding levels are not equal. If they are educated, they say, it is useful and they seek the service but if they are not educated, they say it is not that much important and they don’t seek the service. – IPV survivor, Age 25, Oromiya

Regarding persistent needs for services, survivors emphasized psychosocial counseling. While violence could be temporarily stopped through reconciliation or more permanently through legal actions, trauma from IPV was rarely addressed in either informal or formal IPV support. Many women shared the need for psychosocial services, such as counseling, within more formal IPV support services in order to help women recover from long-term psychological effects of abuse.

There is no organization that gives psychosocial support in this area. We (women) advise each other. – IPV survivor, Age 35, Oromiya

Survivors further emphasized that this counseling should also include concrete solutions to assist in decision-making.

It is good if there are female advisors so that a woman who experiences violence can get this advice immediately; this will help her to calm down and decide the next step. – IPV survivor, Age 28, Oromiya

Lastly, to address unmet need for IPV services due to lack of access, expansion of services, especially women’s affairs offices at the kebele level, was strongly encouraged.

In my opinion, it is better if police station, women’s affair office, court and health center is available at kebele level. Now all these services are not near to us. – IPV survivor, Age 28, Oromiya

Especially if women’s affairs office is opened here, woman who experience violence can access the service without any fear. – IPV survivor, Age 29, Oromiya

## Discussion

Our findings highlight the numerous challenges in help-seeking in both formal and informal systems and the persistent unmet needs of IPV support for survivors, though these needs were not specific to the COVID-19 pandemic. Notably, few women discussed the violence they experienced as unique to pregnancy, with most women referring to IPV over an extended period of time, and both prior to and during COVID-19 restrictions. The majority of IPV survivors in our study heavily relied on their informal network, including, but not limited to, neighbors, village elders, friends, parents, and religious leaders, for protection and assistance in resolving IPV. In Ethiopia, control measures against the COVID-19 pandemic limited social interactions and interrupted public services. Health care facilities resorted to seeing only critical cases, courts reduced the number of sessions, and the police released convicts of petty crimes. Though formal IPV services remained open throughout the pandemic, restrictions left survivors and the larger community with the perception that services were not being provided and this perception may have emboldened perpetrators, while discouraging survivors from seeking help; notably, no survivors described seeking health services during the height of the COVID-19 pandemic.

Consistent with previous studies in Ethiopia and other sub-Saharan African countries, IPV and women’s reluctance in seeking formal services is the norm [14, 18, 23]. For women who did choose to disclose their experiences, they most often chose trusted informal supports within their own families and social networks communities; these findings are consistent with global help-seeking reports and highlight the value of informal supports for women experiencing IPV in LMICs [37]. Specifically, community elders were discussed as the most known and available sources of support, however, the helpfulness of elders varied from highly supportive of women’s goals and offering temporary housing to potentially harmful by prohibiting separation or divorce. While the community elder model has been previously identified as a promising response system to ensure restorative justice, shortcomings that were discussed also include unequal attention to gender power imbalances, with solutions often disproportionately favoring the male partner [38]. Given many women’s trust in informal support systems, including the elder system, and reluctance to involve more formal services, further trauma-informed violence response training for informal resources could help maximize safety and women’s comfort and connection to care, while preserving their rights.

Importantly, COVID-19 control measures affected seeking support from the most common sources—informal IPV resources. Mitigation measures, such as social distancing and intermittent restrictions on public transportation precluded IPV survivors from temporarily seeking refuge during violent situations or reaching out to mediators, such as family or friends, after a violent situation occurred. In line with work from other settings in sub-Saharan African, many women did not want to end the relationship, but preferred to temporarily put space between themselves and their partner [39]; as such, informal sources may serve not only as sources of social support, but also as important safety strategies for IPV survivors seeking to de-escalate violence. Equipping informal sources with trauma-informed bystander tools that provide helpful strategies for listening and responding to IPV survivors, while minimizing shame and blame, may serve as important pathways to enhanced safety decision-making and longer-term safety.

Our study further identified important unmet needs of IPV support in terms of service availability, accessibility, and ease of connection to needed referral services. Survivors did not feel that medical care, psychosocial support, and legal protection, though available at a district level and above, were tailored specifically to IPV survivors needs. Additionally, the services lacked integration, with referral linkages between the different sectors being either weak or nonexistent. Our findings are consistent with Ethiopian stakeholder perspectives, who described a need for coordinated response between response organizations to better support IPV survivors [40]. Fragmented service delivery adds to the complexity of navigating the available resources, impedes access, and limits the utilization of the services [41]. Cognizant of the shortcomings in formal IPV resources, efforts to integrate gender-based violence services are currently underway in Ethiopia [42]. A one-stop-shop approach, where integrated medical, psychosocial, social services, police reporting (if wanted and temporary housing (if needed) are available, is currently being implemented and scaled up across Ethiopia [42, 43]. With this scale-up, innovative approaches will be necessary to meet service demands, as well as ease survivors’ fears and hesitations with seeking formal services.

Distance to resources and access constraints, including transport and money for transport, further emerged as key hindrances to accessing IPV formal services—transport-related issues tied to COVID-19 further exacerbated these barriers. Among other resources, the Ethiopia’s Ministry of Women Children and Youth Affairs provides legal and psychosocial support to IPV survivors at different levels in the administrative structure, however, the closest this office gets to the community is at a district level [43]. In rural Ethiopia, where 85% of the population lives [18], formal IPV resources are found in district capitals that are located far from the majority of their catchment population. While the scale-up of one-stop stop centres throughout Ethiopia remains a potentially promising support service, a broader integration with services closest to women could increase accessibility. For example, further training of Primary Health Care Unit staff, including Health Extension Workers, in basic psychosocial and medical support for gender-based violence, with facilitated referral to more integrated care could assist women in accessing these services within their own communities.

The study is not without limitations. Our purposive sampling of recently pregnant IPV survivors within two regions of Ethiopia allowed for in-depth probing of their recent violence experiences in light of COVID-19 restrictions, however, is not generalizable to all IPV survivors within Ethiopia. Participants of this study were predominantly married women with less than primary level education who lived in rural Ethiopia. These characteristics imply a higher level of violence and limited self-efficacy to respond to violence compared to urban, educated women [44, 45]. Further, our rapid note-taking strategy allowed for rapid, yet thorough, notes without the use of audio recordings to maximize participant confidentiality and ease transcription burden during the height of the COVID-19 pandemic. Quotes are meant to be representative of the conversation and women’s situations but may not be exact quotations nor elucidate further contextual details. While confirmation of the interview notes by both members of the interview team was utilized to ensure women’s stories were accurately portrayed, it is possible that a more comprehensive recording method would have yielded richer quotes and further contextualization to aid in interpretation of findings.

## Conclusion

In conclusion, findings highlight the vast unmet needs of IPV survivors in two regions of Ethiopia, the majority of which precede the COVID-19 pandemic. These results further underscore the importance of women’s empowerment and community awareness programs in alleviating sociocultural barriers to IPV support-seeking. Community-based norms change programs, such as SASA! [46, 47] and Communities Care [48, 49], could serve as important prevention programs to shift gender and power dynamics, as well as norms surrounding shame with disclosure and help-seeking. Safety decision-aids, such as myPlan [50], could further assist with equipping bystanders/informal sources of support with important safety strategies to de-escalate violence through safety plans tailored to women’s situations; such safety decision aids may also be helpful for antenatal or postnatal care to ensure providers have the tools needs to help women enhance safety for themselves and their children. We further urge future public health interventions to strengthen linkages between formal and informal resources to fill the unmet needs of IPV survivors in receiving medical, psychosocial, and legal support in their home communities. These results are timely given the current Ministry of Health efforts to implement one-stop-shop centres across Ethiopia; implementation of these centres is a major IPV response victory for Ethiopian survivors, however, it is clear from these findings that further resources are needed at the community level. Expansion of trauma-informed trainings to enhance screening and care [12] for kebele-level healthcare providers, including health extension workers, with outward, linked referral to higher level care centers could ease accessibility issues, while ensuring that women have necessary access to services, namely, acute medical services for pregnant women experiencing IPV. Lastly, especially in the era of COVID, arranging hotlines and enhancing telehealth will be instrumental for routine IPV screening and providing resources and support to women experiencing violence.

## Data Availability

Given the qualitative nature of this study and to protect participant privacy, data are not publicly available, but are available from the corresponding author upon reasonable request.

## Abbreviations

IPV: intimate partner violence
LMIC: low- and middle-income country
PMA: Performance Monitoring for Action
AAU: Addis Ababa University
FMoH: Federal Ministry of Health
SNNP: Southern Nations, Nationalities, and Peoples’ Region
IRB: Institutional Review Board
